# Understanding How Consumers Categorise Health Related Claims on Foods: A Consumer-Derived Typology of Health-Related Claims

**DOI:** 10.3390/nu11030539

**Published:** 2019-03-02

**Authors:** Charo E. Hodgkins, Bernadette Egan, Matthew Peacock, Naomi Klepacz, Krista Miklavec, Igor Pravst, Jure Pohar, Azucena Gracia, Andrea Groeppel-Klein, Mike Rayner, Monique M. Raats

**Affiliations:** 1 Food Consumer Behaviour and Health Research Centre, Faculty of Health and Medical Sciences, University of Surrey, Guildford, Surrey GU2 7XH, UK; m.egan@surrey.ac.uk (B.E.); m.peacock@surrey.ac.uk (M.P.); n.klepacz@surrey.ac.uk (N.K.); m.raats@surrey.ac.uk (M.M.R.); 2 Nutrition Institute, SI-1000 Ljubljana, Slovenia; krista.miklavec@nutris.org (K.M.); igor.pravst@nutris.org (I.P.); 3 Biotechnical Faculty, University of Ljubljana, SI-1000 Ljubljana, Slovenia; Jure.Pohar@bf.uni-lj.si; 4 Centro de Investigación y Tecnología Agroalimentaria de Aragón (CITA), 50059 Zaragoza, Spain; agracia@aragon.es; 5 The Institute for Consumer & Behavioural Research, Universität des Saarlandes, D-66123 Saarbrücken, Germany; groeppel-klein@ikv.uni-sb.de; 6 British Heart Foundation Centre on Population Approaches for Noncommunicable Disease Prevention, Nuffield Department of Population Health, University of Oxford, Oxford OX3 7BN, UK; mike.rayner@dph.ox.ac.uk

**Keywords:** nutrition claims, health claims, consumer understanding, multiple sort procedure, health claim typology

## Abstract

The Nutrition and Health Claims Regulation (NHCR) EC No 1924/2006 aims to provide an appropriate level of consumer protection whilst supporting future innovation and fair competition within the EU food industry. However, consumers’ interpretation of health claims is less well understood. There is a lack of evidence on the extent to which consumers are able to understand claims defined by this regulatory framework. Utilising the Multiple Sort Procedure (MSP), a study was performed (*N* = 100 participants across five countries: Germany, the Netherlands, Slovenia, Spain and the United Kingdom) to facilitate development of a framework of health-related claims encompassing dimensions derived from consumers. Our results provide useful insight into how consumers make sense of these claims and how claims may be optimised to enhance appropriate consumer understanding. They suggest consumers may not consciously differentiate between a nutrition claim and a health claim in the way that regulatory experts do and provide insight into where this might occur. A consumer-derived typology of health-related claims based on three key dimensions is proposed: (1) Familiarity with the nutrient, substance or food stated in the claim; (2) statement type in terms of simplicity/complexity; (3) relevance of the claim, either personally or for a stated population group.

## 1. Introduction

Noncommunicable diseases (NCDs) are the primary causes of death and disability [1] and risk factors associated with unhealthy diets contribute to the development of NCDs such as obesity, type 2 diabetes and cardiovascular disease. The provision of information on packaged processed foods is seen as having the potential to support informed choice by the consumer in the food domain [2], whilst not curtailing the food industry’s freedom in terms of the products they produce [3]. With the mandatory introduction of nutrition labelling for all packaged foods required by 2016 [4], the concept of informed choice by consumers within the food domain has become synonymous with health policy related to encouraging consumers towards a healthier choice. Often seen as complementary to mandatory nutrition labelling, nutrition and health claims are a means by which health-related information may be communicated to the consumer on food packaging. However, the plethora of nutrition and health claims appearing on food products in Europe was seen a cause for concern due to the lack of harmonised approach to the substantiation of such claims and their potential to mislead the consumer [5]. 

Implementation of The Nutrition and Health Claims Regulation (NHCR) EC No 1924/2006, initially sought to eliminate unsubstantiated and potentially misleading claims from the marketplace and create a regulatory framework which would provide an appropriate level of consumer protection, while also supporting future innovation and fair competition within the EU food industry [6]. The NHCR legislation requires all claims, implying a health benefit of consuming a food, to be substantiated by a review of the scientific evidence and included on the list of authorised health claims in the European Union (EU) Register [7], before being used on a food product. The legislation primarily focuses on text-based claims, but also refers to pictorial, graphical or symbolic messages which state or imply that a food has particular nutritional or health benefits. For the purposes of implementing and enforcing the regulation, health claims are more specifically defined by three main categories: ‘general function’ or Article 13 claims, ‘risk reduction’ or Article 14(1)a claims, and claims relating to ‘children’s development’ or Article 14(1)b claims. All new claims need to be submitted for approval via an application dossier before they can be used on food products. The regulation also specifies that the use of a nutrition and health claim is only permitted if the average consumer can be expected to understand the beneficial effects expressed in the claim [6]. 

Although the NHCR has exercised control over the way in which claims are presented by manufacturers on pack, consumers’ interpretation of those claims is less well understood and there is a lack of evidence to establish the extent to which the average consumer is really able to understand health claims [8,9]. In addition, Roe, Levy & Derby (1999) proposed that the presence of health claims may under some conditions lead to situations whereby incorrect inferences are made about a product by the consumer [10]. In their paper on the determinants of consumer understanding of health claims, Grunert, Scholderer and Rogeaux suggested that previous research on consumer understanding in this domain has failed to effectively measure understanding of health claims in terms of inference-making [8]. It has also been demonstrated that consumers may not understand health and nutrition claims in the way in which the NHCR intends [8,11,12] and tend to interpret them differently from scientific experts and regulators [13,14]. This raises questions about how relevant the expert categorisations utilised in the NHCR regulation are from a consumer perspective. 

Utilising Multiple Sort Procedure (MSP) methodology, this study seeks to (1) help develop a framework of nutrition and health claims considering the point of view of consumers and the way they make sense of this information, and following this (2) identify how claims may be optimised to favour an adequate understanding and use of this information.

## 2. Materials and Methods 

### 2.1. The Multiple Sort Procedure (MSP) and Multiple Scalogram Analysis (MSA)

The importance of categorisation is well established in the field of psychology [15]. ‘The Multiple Sort Procedure (MSP)’ [16,17] is a form of facet theory and involves ‘free sorting’ of a range of stimuli facilitating a systematic exploration of the various ways in which participants’ make sense of a particular topic. Eliciting the descriptive terms or constructs derived by an individual, or group of individuals when freely categorising a range of stimuli, facilitates the development of a conceptual framework of a domain to be created. The value of this conceptual framework is that it is formed by a bottom-up approach, not constrained by the application of a priori framing, and is therefore more likely to lead to a deeper understanding of how a particular domain is really conceptualised by the group of interest. In addition, it is possible to inform further the conceptual framework derived from free sorting by imposing predefined categories in the form of a ‘structured sort’. In this way, different dimensions and conceptual frameworks can be compared and explored. In their review on consumer understanding of claims and appropriate methodologies, Leathwood et al. (2007) highlight the usefulness of qualitative methodology as an important step towards gaining deeper insight into consumer understanding of health claims, but stress the need to avoid predefined concepts within this research area [11]. MSP has previously been used in a number of domains including environmental, social and criminal psychology where elicitation of constructs, pertinent to understanding these domains, from the group of interest, is considered more valid than the use of constructs predefined by the experts [18,19]. More recently it has been successfully employed to develop a consumer-derived typology for front-of-pack nutrition labelling [20]. 

Whilst MSP is essentially a qualitative method, the underlying structure of the qualitative data generated can be explored using Multiple Scalogram Analysis (MSA) [21,22,23]. MSA is a statistical procedure that produces a scatter plot depicting each item as a point in two-dimensional space, based on the way in which the item was assigned to categories in the sorting process. The spatial proximity or distance between the points on the plot reflects the overall conceptual similarity or difference of the sorted items [17]. By overlaying the qualitative data onto the plot, the items being studied can be partitioned into meaningful regions by the researcher and then used to inform the development of typology of the domain.

### 2.2. Procedure

Our study involved both free and structured sorting of a range of health and nutrition claim statements presented on cards. The underlying structure of the qualitative data generated by the free sorting was then explored using Multiple Scalogram Analysis (MSA). The structured sorting data was explored in terms of frequencies and the qualitative data used to facilitate interpretation of the frequencies observed.

Individual face-to-face interviews were conducted using a standardised interview schedule. Participants were given a set of twenty-five cards, each of which displayed a claim statement, and were instructed to free sort the cards into groups so that all the cards in one group were similar to each other in some important way and different from the other groups. They were then asked to repeat this using a different sort rationale if they could. Participants were encouraged to ‘think aloud’ both about the cards and their sorting rationale (free sorting). Next, participants were asked to sort the same set of stimuli cards into groups against preassigned expert typology headings which reflected the NHCR framework (structured sorting). The interviews were audio recorded, and the interviewer made note of the overall sort rationale used by the participant, the reasons for each grouping of cards and which cards were assigned to each group. Throughout the sorting activities, the interviewer prompted the participant to ‘think aloud’, but did not engage in discussion about the health claims themselves or where they should be placed. Finally, the participant completed the self-report background questionnaire and received a debriefing about the study. 

This study received a favourable ethical opinion from the University of Surrey Ethics Committee for the UK and Netherlands data collection and was approved according to local practices by the University of Saarland, Agrifood Research and Technology Centre of Aragon and the University of Ljubljana for the German, Spanish and Slovenia data collection. All subjects gave their informed consent for inclusion before they participated in the study. 

### 2.3. Participants

The study involved a total of 100 participants, who shopped for groceries at least occasionally and comprised of 20 participants from each of the following countries; Germany (DE), the Netherlands (NL), Slovenia (SL), Spain (ES) and the United Kingdom (UK). Recruitment quotas were applied for sex, age and highest education level in each country. The resultant sample profiles by country are detailed in Table 1. 

### 2.4. Rationale for the Development of the Study Stimuli

The claim statements used as stimuli in the sorting tasks were selected to represent a range of General Function Claims, Disease Risk Reduction Claims, Nutrition Claims and claims from the General Health Claims category. In the selection process the following criteria were taken into consideration;

- General function claims (13(1) a–c) relating to a range of different nutrients, substances, food or food categories and representing a range of different health relationships. The wording for these was taken verbatim from the EU Register of Nutrition and Health claims [7].
- Disease risk reduction/children’s development and health claims (14(1) a–b) again, where possible, relating to a range of different nutrients, substances, food or food categories and health relationships. The wording was taken verbatim from the EU Register of Nutrition and Health claims [7].
- Nutrition Claims for nutrients or ingredients (i.e., nutrient content claims related to Vitamin C, Sodium, Fat, Sugar and Omega3).

**Figure 1:**
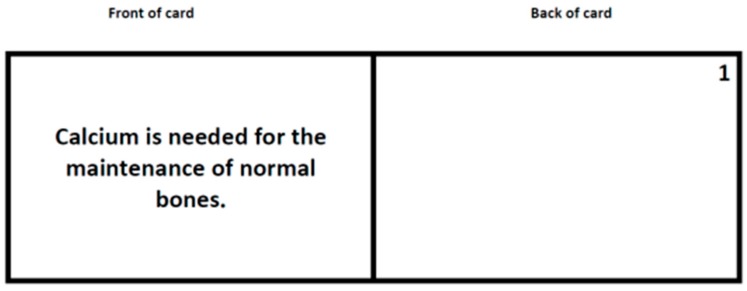
Example of stimuli card number 1 utilised in the study.

Finally, two claims were included which are classified by some experts as General Health claims because of the health relationship implied by the ingredient over and above that of a simple nutrient content claim, but are in contrast considered as nutrient content claims by other experts due to the lack of a stated function or benefit in the claim. These were ‘Contains wholegrain’ and ‘One of your 5 a day’. Due to the lack of familiarity with the ‘One of your 5 a day’ claim in some of the study countries, a short explanatory statement was added to the stimuli card; ‘Experts recommend you eat 5 portions of fruit and vegetables every day. That is 5 portions in total, not 5 portions of each’. The maximum number of stimuli that a participant can realistically process within a study of this type is typically between fifteen and twenty five separate elements [17,24], for this reason, a limit for the stimuli was set at twenty-five. An example of one of the stimuli cards used in the study can be seen in Figure 1. The wording of the claim stimuli and associated expert typology category are shown in Table 2.

### 2.5. Structured Sorting—Expert Typology Headings

The expert typology headings utilised in the structured sort were derived directly from the current EU regulation 1924/2006 [6] resulting in five group headings pertaining to health claims and one group heading pertaining to nutritional claims (Table 3). A further group heading simply entitled ‘Don’t know’ was provided for use when participants were unable to place a particular stimuli card under any of the other six expert typology headings. 

### 2.6. Background Measures

Participants were required to self-complete a questionnaire to provide information on behaviour and attitudinal variables. All were measured on five-point Likert scales. The questionnaire comprised eight General Health Interest items [25,26], three items relating to subjective health claim knowledge adapted from Moorman et al. (2004) [27] and two motivation to process health claims items [28]. Processing style, useful for understanding how individuals attend to and interpret components of a given message, was measured with short versions of the ‘Need for Cognition’ [29] and ‘Faith in Intuition’ scales [30]. Usage of health claims was measured by the item ‘I often use health claims and symbols on food in general while shopping’. The questionnaire was developed in English and translated with some minor country-specific modifications to make it appropriate across all four countries.

### 2.7. Free sort Analysis

Multiple Scalogram Analysis [21,23,24] involves the preparation of a data matrix in which each column represents an individual participant’s sort, and each row represents a card, that is, a particular claim. The Multiple Scalogram Analysis (MSA) provides an overall ‘top’ plot that depicts the relationships between all the cards in that analysis. Each card is a point in geometric space and the closer the points are to each other, the more similar they are considered to be by the participants. The program imposes a ‘coefficient of contiguity’ of 0.9 minimum to ensure that the solution being produced is an acceptable fit to the data. Regardless of whether differing numbers of categories were used by the participants during their individual free sorts, the cards that were most frequently placed together across the whole sample appear closest together on this top plot. 

In addition to this top plot, the MSA output also includes an ‘item’ plot for each sort included in the data matrix. The configuration of the points on these item plots is the same as for the top plot, however, this time the points represent the category or group as described by the participant. The qualitative data captured during the sorting activities; category descriptions assigned by each participant, their explanation as to why cards assigned to a given category were considered similar to each other/different from cards in other groups and any specific comments they made about particular cards were overlaid onto these item plots. A content analysis of the qualitative data across the whole sample was also conducted to provide an overview of the constructs participants used to describe their sort groupings and reasoning and these were subsumed into broader thematic categories (Appendix A Table A1 and Table A2). These categories were then used in conjunction with the individual constructs to facilitate the interpretation of the MSA top plots. In this way, the researcher was able to partition the top plot on the basis of why particular cards were put together, and offer an interpretation of the categories that have informed the way in which the study participants have sorted the cards [17]. 

Plots of the first free sorts for each country were prepared as a starting point for the analysis and each country analysed separately enabling exploration of the differences between countries. 

### 2.8. Structured Sort Analysis

The ability of participants to assign the stimuli cards to the appropriate structured sort heading groups was established via frequencies and the qualitative data used to facilitate interpretation of the frequencies observed. The purpose of this task was to develop an understanding of where there might be differences between how consumers perceive the claims presented to them, compared to experts. 

## 3. Results 

### 3.1. Sample Description 

Overall, 82% of the sample described themselves as being either the main shopper or shopping as frequently as someone else in their household for food products: UK 85%, NL 95%, DE 75%, SL 75% and ES 80%. In terms of use of text or image-based health claims on food products when shopping, 51% of the sample described themselves as using them either “Quite often” or “Very often”, with 48% using them “Sometimes” or “Rarely” and only 1% stating that they never used health claims when shopping. 

There were no significant differences between countries in terms of participants’ self-reported General Health Interest, Need for Cognition, Faith in Intuition or subjective knowledge of health claims (Table 4). However, post-hoc tests revealed a significant difference between Spain and the Netherlands in terms of their motivation to process health claims. Spain reported the highest motivation to process health claims, with the Netherlands reporting the lowest, and the UK, Germany and Slovenia falling in between.

### 3.2. Constructs Utilised by Participants in Their First Free Sort

An overall frequency table was prepared for the constructs utilised across all five countries by the participants in all their free sorts for the category/group labels (Appendix A Table A1) and a table reflecting the frequency of constructs used in the first free sorts per country (Appendix A Table A2). Interview transcripts and overall sort criteria were used to guide interpretations of the group labels with similar meanings. Most participants managed to do at least two sorts, 40% managed three sorts and 9% managed to do five sorts. A total of 245 free sorts were recorded across the whole sample (*N* = 100).

Overall, a total of 17 categories of constructs were used across all the countries and, of these, 13 were used in three or more countries. The most frequently utilised constructs related to participants’ attempts to sort the cards based on the information contained within the claim:

- Nutrient, health condition or outcome, function and/or purpose of the claim.
- Types of statements in terms of their complexity, length or levels of information.
- Relevance of the claim to the participant personally or their ability to see a claim’s relevance to a specific population group.

### 3.3. First Free Sort Top Plots

Top plots of the first free sorts for each country were prepared as a starting point for the analysis. The individual plots were initially interpreted at country level in relation to the constructs used, and the additional qualitative data gathered and then compared/contrasted between countries. In addition to running the MSA analysis on the first free sorts, top plots were generated for the second free sorts for each country. The frequency at which new constructs appeared in subsequent free sorts was quite low, plots for these did not appear to add any different dimensions to the interpretations already provided, and further analysis was not pursued. 

#### 3.3.1. Top Plot—United Kingdom (UK)

**Figure 2:**
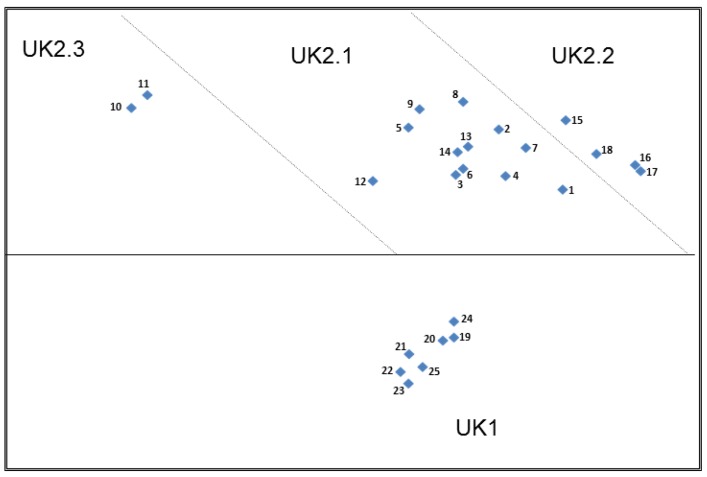
Multiple Scalogram Analysis (MSA) Top Plot—United Kingdom (UK). See Table 2 for the claims associated with the stimuli card numbers.

By analysing the qualitative data provided by the UK participants for the sort strategies, the group headings assigned and their comments about specific cards during their sorting activities; the UK plot (Figure 2) was partitioned in to two main groups. The first group, UK1 consisted of Cards 19–25: the nutrition claims. The second group, UK2 consisted of all the health claims: Cards 1–18. UK2 was then further partitioned into three subgroups. 

The partitioning between the main UK1 area and UK2 related to the difference between the types of statements in terms of their simplicity/complexity and information levels, but also to the difference between the two partitioned groups with respect to the presence/absence of a stated function/benefit or purpose. UK1 contained the shorter, simpler nutrition claims that did not contain a stated function or benefit and UK2 contained the more complex claims with higher levels of information. UK2 could be further subdivided in terms of the construct of ‘relevance’ whereby the cards in subgroup UK2.2 and UK2.3 appeared relevant for those on a weight loss diet or for children, but those in subgroup UK2.1 were described more generally as not personally relevant or participants were unsure for whom they would be relevant.

In addition to the partitioning described above, there was a directional element to the overall UK plot, in that those cards appearing towards the top of the plot were referred to as less understandable than those appearing towards the bottom, regardless of which subgrouping of UK2 that they were placed in. For example, Cards 10, 11, 8, 9 and 15 appearing at the top of the plot contained unfamiliar nutrients or terms such as ‘Glucomannan’, ‘Meal replacement’, ‘Pantothenic acid’ and ‘Docosahexaenoic acid’ (DHA) which participants described negatively in terms of understanding. Conversely, claims appearing at the bottom of the UK2 partition contained the more familiar macro and micronutrients typically found in the more familiar nutrition claims. This theme is also the most likely explanation for the distance between Cards 15 and 18 from Cards 16 and 17 in UK2.3.

“The DHA I mean, I don’t know that one, and there the DHA is on there again. I don’t… obviously it’s something important but I don’t really understand enough to understand…. what it’s significance is.… but I’d have to go back and put that into Google, because I don’t know what that is”.

(Cards 8 and 15, UK)

“I would look at that and think I don’t know what the damn Pantothenic acid is! So I wouldn’t know whether it’s going to do me any harm or good or whatever”.

(Card 9, UK)

Participants’ expressed a lack of understanding/familiarity with the nutrient ‘DHA’ and a lack of familiarity with ‘essential fatty acids’ when compared to the more familiar nutrients such as calcium, vitamin D and iron which were more easily recognisable by the participants as being beneficial for children.

“I didn’t know that they [children] needed fatty acid”.

(Card 18, UK)

“Fatty acids for growth and development? Not really”.

(Card 18, UK)

A number of participants expressed concerns regarding the credibility of the weight loss claims and many found them difficult to understand or simply didn’t agree with them. This explains their positioning towards the top of the plot and possibly also their extended distance from the other clusters within UK2.

“Substitute one meal on an energy restricted diet? See, I don’t ever think that’s good. A meal replacement. Would that be a meal replacement drink? See, I don’t ever agree with them”.

(Card 10, UK)

“Substituting one meal a day for an energy… I don’t think that’s good for you. I don’t believe in- well, I wouldn’t do that anyway….substituting one of your meals, unless your physician had told you to do that, I wouldn’t be very happy with that”.

(Card 10, UK)

“I don’t really understand what that’s trying to tell me.... I am on a diet, but I kind of feel like that might be something that.... not really sure about that... bit suspicious, yes”.

(Card 11, UK)

Some participants were sceptical about the sugar-free gum health claim (Card 12), which may explain its distance from the main cluster of health claims in UK2.1 and its proximity to the weight loss claims in UK2.3 rather than the other health claims in either UK2.1 or UK2.2.

“It’s like they’re trying to find something good for something that isn’t necessarily – do you know what I mean? It’s chewing gum! It’s not like a food thing, you know. Oh yeah, they’re trying to make out its good for your teeth, which is probably true but I wouldn’t expect it on a food type thing”.

(Card 12, UK)

The nutrition claims in UK1, were generally described more favourably because of the simplicity of the statements although it was recognised that these claims lacked a function/benefit or purpose in the claim statement and these two constructs appear to explain why these claims are separated from those in UK2. The UK participants did express some concerns about the credibility of many of the nutrition claims particularly those relating to fat (Card 21) and sugar (Card 22), but, since this cluster of claims is quite compact on the MSA plot, this construct did not appear to be reflected in their sorting strategy.

“I’m a bit dubious about “fat-free” because there are different kinds of fat”.

(Card 21, UK)

“No added sugar? I’m not sure about that. Does it mean that it’s already got a load of sugar but they’ve not added anymore?”

(Card 22, UK)

#### 3.3.2. Top Plot—Germany (DE)

**Figure 3:**
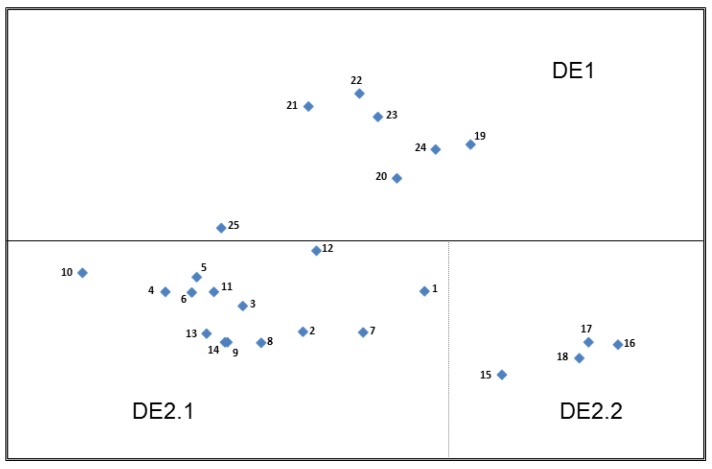
MSA Top Plot—Germany (DE). See Table 2 for the claims associated with the stimuli card numbers.

Overall, there is more separation and less defined clustering when comparing the German MSA plot to the UK plot but two main partitions were identified (Figure 3). DE2 contains the more detailed health claims and DE1 contains the simpler nutrient claims and the construct of ‘Types of statements’ is again driving the overall positioning of the points on the plot. DE2 could be further subdivided in terms of the construct ‘relevance’, with those claims specifically relevant for children in subgroup DE2.2 and those that were not in subgroup DE2.1.

The increased distance of Card 15 from the other cards in subgroup DE2.2 is again most likely to relate to the presence of the unfamiliar nutrient ‘Docosahexaenoic acid’ (DHA) in the claim. Again, when compared to the more familiar nutrients such as calcium, vitamin D and iron, it was less easily recognisable by the German participants as being beneficial for children.

“Because I don’t know what DHA means…. I never heard about it, so I don’t know what to think about it”.

(Card 15, DE)

“DHA means nothing to me…acid means nothing to me”.

(Card 15, DE)

The proximity of Card 1 ‘Calcium is needed for the maintenance of normal bones’ to DE2.2 is most likely due to some participants’ recognition that calcium is important for children.

“Children also need calcium and vitamins for their bones and other things”.

(Card 1, DE)

The distance between Card 10, the meal replacement claim and the remainder of cards in subgroup DE2.1 is most likely explained by some participants’ expressed dislike or disbelief associated with this claim.

“I do not like the idea of meal replacements because I really like to eat. I think of some disgusting barley-based drinks and cannot really make any use of it”.

(Card 10, DE)

“That’s nonsense, as well as weight loss drinks, that is made-up. I don’t believe it!”

(Card 10, DE)

On the whole, the sugar-free gum claim, Card 12, received a more favourable response, but due to its perceived relevance to either weight loss, children or the fact that some participants did not feel that dental caries was in fact a disease, this card is slightly separated from the main cluster of other health claims within DE2.1.

“Sugar-free chewing gum is more for children or teenagers because adults do not chew gums”.

(Card 12, DE)

“If you want to lose weight, you should not consume any sugar. A sugar-free chewing gum can help”.

(Card 12, DE)

“Caries is not directly a disease. But this one with the sugar-free chewing gum is a kind of advice”.

(Card 12, DE)

With respect to the nutrition claims in DE1, there is more distance generally between the cards in this grouping when compared to the UK plot, but particularly for Card 25 ‘One of your 5 a day (Experts recommend you eat 5 portions of fruit and vegetables every day. That is 5 portions in total, not 5 portions of each)’ which is closer in proximity to the DE2.1 group than the other nutrition claims. This may be due to the length of text on the claim when compared to the other nutrient claims which tended to be more concise, but it may also be due to the recognition by some participants that for them this claim implies health perhaps more so than the other nutrition claims included in DE1.

“This information is also easy to understand. It is an indication that it is definitely healthy, because they suggest it”.

(Card 25, DE)

The remaining nutrition claims were, in general, thought to be more related to the product itself and what it contains:

“Fat-free, contains whole grain, no added sugar—everybody can understand it. These are statements which have a meaning. They are short. The statements are striking; they tell you something about the product. In my opinion, they belong on the front of the pack”.

(Card 21–24, DE)

“Rich in vitamin C, contains wholegrain, no added sugar, I think that these are some keywords about the content of the product. These are claims about what it contains, not recommendations per se”.

(Cards 19–23, DE)

There was some confusion regarding the sodium (Card 20) and omega 3 (Card 24) claims in relation to whether these were, in fact, positive nutrients or not and a few participants expressed scepticism about the fat and sugar claims (Cards 21 & 22), although this was not a dominant theme in the German sample.

#### 3.3.3. Top Plot—The Netherlands (NL)

**Figure 4:**
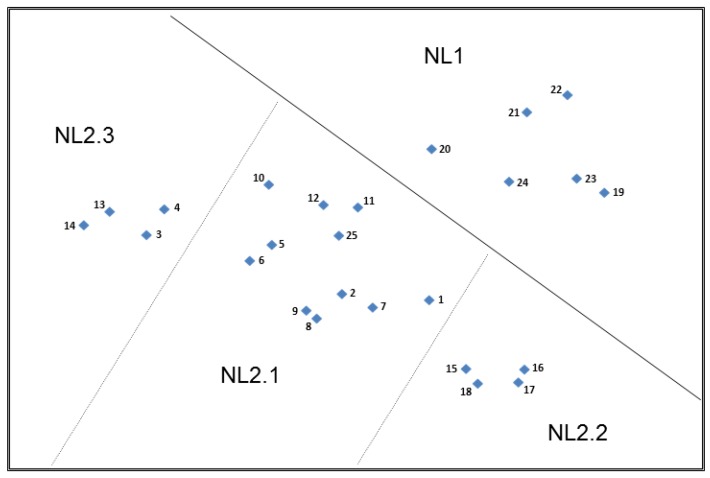
MSA Top Plot—The Netherlands (NL). See Table 2 for the claims associated with the stimuli card numbers.

Similarly to the UK and German plots, the Dutch plot reflected two main partitions; however, this time the main partitioning related to the construct of ‘Information contained in the claim’ (Figure 4). Following the pattern of the UK and German plots, NL2 contained the more detailed health claims with higher levels of information content and NL1 the shorter, more simplistic nutrition claims, but sorting was driven more by the presence/absence of a stated function/benefit or purpose as the dominant theme. 

NL2 could be further subdivided into three groups and the construct driving the separation of the cards in NL2 related to the health condition/function or purpose of the claim. The cards in subgroup NL2.2 were easily recognisable by participants as relating to children’s growth and development and those in subgroup NL2.3 appear close together due to their common relationship with blood pressure and heart health. The remaining claims in NL2.1 contained all the other general function and disease risk reduction claims.

It is perhaps interesting to reflect at this point on why further separation was not observed between the claims in NL2.1. For example, one might also have expected a cluster relating to weight loss claims or perhaps a cluster relating to psychological or behavioural functions. It is possible that due to a lack of familiarity with the stated nutrient, a lack of understanding of the stated function and/or differences with respect to perceived credibility of the claims in this main grouping, these claims were not consistently sorted by the participants and therefore no clear clustering exists within this subgroup. This perhaps also explains why the claim related to ‘One of your 5 a day’ exists in this group.

“Those five times a day [fruit and vegetables], I cannot quite place”.

(Card 25, NL)

“One of your 5 a day—that is a general health advice”.

(Card 25, NL)

#### 3.3.4. Top Plot—Slovenia (SL)

**Figure 5:**
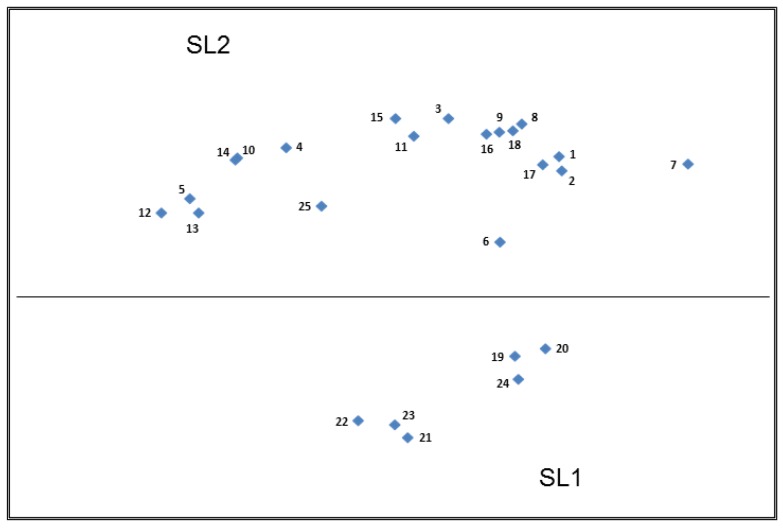
MSA Top Plot—Slovenia (SL). See Table 2 for the claims associated with the stimuli card numbers.

The Slovenian plot (Figure 5) could be partitioned into two main groups with SL1 containing the shorter, simpler nutrition claims and SL2 containing the more detailed health claims. The construct of ‘Types of statements’ dominated the overall positioning of the points on the plot. However, due to the wider variety of sort strategies utilised by the Slovenian participants when compared to the UK, Germany and The Netherlands, further partitioning of the claims within SL2 was not possible as no consistent construct appeared to dominate the observed separation. 

Some Slovenian participants utilised a sort strategy relating to the food relevance of the claim, this construct did occur in some of the other countries in their subsequent sorts but at a relatively low frequency.

“Sodium? I would position it to the meat group. Each meat has at least a bit of sodium”.

(Card 3, SL)

“This acid (DHA) can be probably found in fruits or vegetables”.

(Card 8, SL)

“Zinc—vegetables contain plenty of it, spinach I think, if I am not mistaken”.

(Card 7, SL)

“Omega 3 are in fish”.

(Card 24, SL)

Whilst there appears to be two distinct clusters of cards within SL1 on the Slovenian plot, no consistent explanation for this could be found in the qualitative data or sorting strategies. Some Slovenian participants had difficulty understanding the less familiar nutrients in Cards 19, 20 and 24,i.e., ‘Rich in vitamin c’, ‘Naturally low in sodium’ and ‘Source of omega-3’, respectively, and this may have contributed to the separation between these two clusters. Despite the lack of sub-partitioning within SL2, there were similarities with the other countries in the way in which some of the Slovenian participants described certain health claims, with those containing unfamiliar or scientific nutrients, substances or functions being less favourably received.

“DHA—I don’t know what this is; it must be more of a technical term”.

(Card 8, SL)

*“I don’t know what this* [pantothenic] *acid means”.*

(Card 9, SL)

“Glucomannan? I don’t know it! I know glucosamine, for cartilage”.

(Card 15, SL)

“If only I knew what homocysteine metabolism is, I have no idea, first time hearing”.

(Card 2, SL)

The Slovenian qualitative data also echoed that seen in the UK sample regarding the sugar-free gum claim.

“Chewing gun is just chewing gum, it is not food”.

(Card 12, SL)

“Chewing gum in my opinion is not important for health; this is my opinion probably based on the fact that I am not an admirer of chewing gum”.

(Card 12, SL)

Similarly to in the Dutch plot, Card 25—‘One of your 5 a day’—appears on the Slovenian plot within the SL2 partition, that is, with all of the health claims as opposed to with the nutrition claims. As per the German plot, this may in some part be due to the length of text on the claim when compared to the other nutrient claims. However, the qualitative data suggest that it may again be due to the recognition by some participants that this claim implies health perhaps more so than the other nutrition claims included in SL1.

*“Let put into this group* [group named important for health] *also 5-a-day regarding that we have ‘succumbed’ to commercials”.*

(Card 25, SL)

“5 a day, reminds me of healthiness, not being fat, healthy lifestyle...”

(Card 25, SL)

#### 3.3.5. Top Plot—Spain (ES)

Though participants across all the countries found free sorting these types of stimuli difficult, which resulted in a number of mixed sorts with no dominant construct, this was most prevalent in Spain. Here, seven of the twenty participants’ first free sorts resulted in a mixed sort, and of these seven participants, four were unable to do any further sorts. 

**Figure 6:**
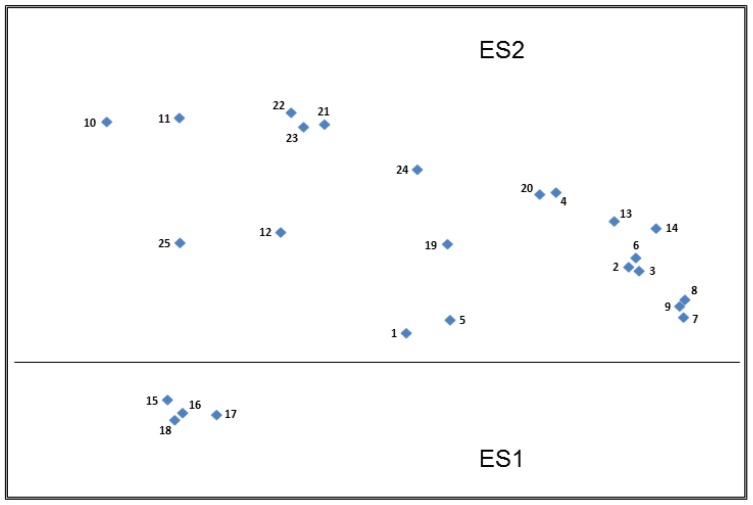
MSA Top Plot—Spain (ES). See Table 2 for the claims associated with the stimuli card numbers.

Again, similarly to the Slovenian plot, the Spanish plot demonstrated two main partitions (Figure 6) and it is apparent from the close clustering of the cards in ES1 (Cards 15–18), that the Spanish participants frequently placed these together in their sorting. The qualitative data suggests that this is due to these claims being easily recognisable as being relevant for children. 

For all remaining claims in ES2, no dominant constructs could be applied to partition the group any further, implying that similarly to Slovenia the Spanish participants had more of an individualistic approach to sorting the claim stimuli than seen in the UK, Germany or The Netherlands. This individualistic approach appeared to be driven by personal interest in health or the relevance of particular claims to them based on their experiences of having family members with particular food-related health conditions. Despite this, the qualitative data suggests that the Spanish participants were utilising similar constructs to those seen in the other countries, namely those relating to the information contained in the claim, the types of statements in terms of their complexity/simplicity and their relevance to either themselves as an individual or other specific population groups. In addition, the Spanish participants expressed similar difficulties with understanding or accepting claims which contained unfamiliar nutrients or functions:

“I do not know what homocysteine is”.

(Card 2, ES)

“DHA, I do not understand the meaning”.

(Card 8, ES)

“Pantothenic acid, I do not understand the meaning”.

(Card 9, ES)

“Glucomannan, I do not understand”.

(Card 11, ES)

There was also the perception that the shorter, more simplistic nutrition claims were easier to understand than the more detailed health claims. The Spanish participants also recognised that they differed in terms of the presence/absence of a stated function or benefit.

“Some cards have short sentences easier to understand”.

(Cards 19–24, ES)

“Information about the benefits for the human health”.

(Cards 5, 6, 10 & 12, ES)

“Inform about the substances that the food contains but it does not mention the benefits of the substance for the human health”.

(Cards 19, 20, 24 and 25, ES)

“This information does not say the benefits it provides”.

(Card 25, ES)

### 3.4. Structured Sorting into Expert Group Headings

#### 3.4.1. Placement of Article 14a and 14b Claims

**Figure 7:**
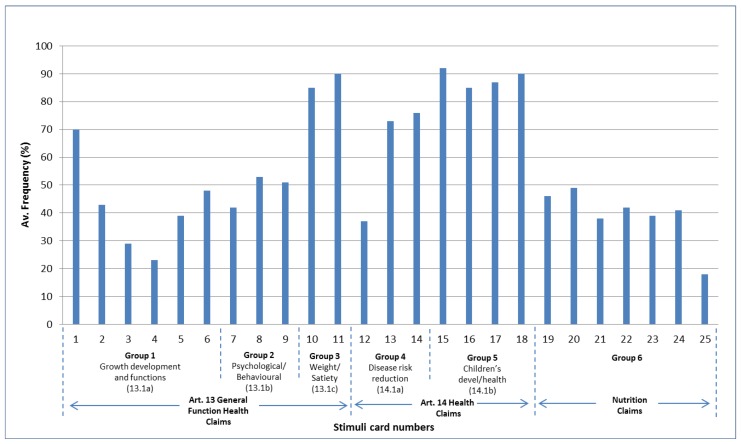
Average frequency (%) of placement in appropriate structured sort groups across all countries. See Table 2 for the claims associated with the stimuli card numbers.

Across the whole sample (*N* = 100), most of the participants were able to assign the Article 14a disease risk reduction (Cards 13 and 14) and Article 14b children’s development and health claims (Cards 15–18) to their appropriate expert typology structured sort groups (Figure 7). Due to the inclusion of the disease risk reduction element of the claim in the claim statement, or the fact that the children’s claims clearly stated that they related to children, on the whole it was clear to participants where these claims should be placed. However, Card 12, ‘Sugar-free chewing gum helps reduce tooth demineralisation. Tooth demineralisation is a risk factor in the development of dental caries’ was an exception within the Article 14a claims, with less than 40% of the total sample assigning this card to its appropriate expert typology group. Some participants were unable to accept sugar-free gum as a food, others did not recognise dental caries as a disease risk factor, despite this being stated in the claim, and therefore these participants experienced difficulty in placing the card. Other participants felt that sugar-free gum most closely related to children and therefore assigned this claim to expert typology Group 5.

“Caries is not directly a disease …this one with the sugar-free chewing gum is a kind of advice”.

(Card 12, DE)

“I will place sugar free chewing gum by the cards about children”.

(Card 12, NL)

“Dental caries – I don’t know why, but I don’t think adults have it”.

(Card 12, SL)

“And here is a claim about sugar-free gum, but why do you need to have sugar free gum in the first place is my question”.

(Card 12, UK)

#### 3.4.2. Placement of Article 13 Claims

The two Article 13c weight control/satiety claims (Cards 10 and 11) were assigned to their appropriate structured sort group by the majority of participants (Figure 7). This appeared to be driven by the fact that participants could directly associate these claims with weight control either as a function or as being relevant for a target group (i.e., for people on a weight loss diet) and, therefore, assigned them appropriately to Group 3.

With the exception of Card 1 ‘Calcium is needed for the maintenance of normal bones’, the remaining Article 13 claims posed more of a challenge for participants with Cards 8 and 9 only being assigned to their appropriate group by approximately half the sample and just over 40% of the sample for Cards 2 and 7. The qualitative data suggested that some participants were unfamiliar with the meaning of the term ‘cognitive function’ in Card 7 and this made it difficult for them to assign this card to its appropriate group. Claims relating to ‘brain function’ in Card 8 and ‘tiredness and fatigue’ in Card 9 appeared to be more easily recognisable as being related to ‘psychological and/or behavioural functions’.

“I don’t know what ‘cognitive’ means”.

(Card 7, DE)

“Zinc contributes to normal cognitive function… cognitive means mind, doesn’t it?”

(Card 7, SL)

“What are cognitive functions? I mean, zinc is important for the body, I know this. But I don’t know what to think about this term, this function”.

(Card 7, DE)

Some participants did not understand or recognise the function ‘homocysteine metabolism’ in Card 2 and tended to place this card in the ‘Don’t know’ group (Appendix A, Table A3) rather than Group 1. 

Of the remaining Article 13a claims ‘Reducing consumption of sodium contributes to the maintenance of normal blood pressure’ (Card 3) and ‘Replacing saturated fats with unsaturated fats in the diet contributes to the maintenance of normal blood cholesterol levels’ (Card 4), were almost as frequently placed in Group 4, the disease risk reduction group, as in the appropriate Group 1 general function claims group heading. This suggests that despite these claims only referring to maintenance of normal blood cholesterol they were being perceived as more relevant to the disease risk reduction group.

“Consumption of sodium… It helps to reduce a disease for people with high blood pressure”.

(Card 3, DE)

“If you use too much salt, it raises your blood pressure”.

(Card 3, SL)

“Improvement of the overall condition by unsaturated fats”.

(Card 4, DE)

The claim regarding live cultures improving lactose digestion, Card 5 was only placed in the appropriate expert typology Group 1, by 39% of the total sample. Some participants experienced difficulties placing this card as either they did not understand the claim, or they did not recognise it as being relevant to them so put it in the ‘Don’t know’ Group 7. Others felt that lactose intolerance was some form of disease and therefore placed it in Group 4.

“I don’t have lactose intolerance. I don’t know where it belongs”.

(Card 5, DE)

‘’Live cultures improve digestion—well, I do not believe this, and we have never learnt about this in school. This must be yoghurt commercial”.

(Card 5, SL)

“This is about a kind of disease”.

(Card 5, NL)

“This is a strange claim. It is for people who are sick”.

(Card 5, NL)

Conversely, other participants placed Card 5 in Group 6 in recognition of the beneficial properties of milk/yoghurt for them, their belief that this claim isn’t related to disease and that those affected simply needed to eliminate it from their diet.

“In my opinion, lactose intolerance is not a huge disease…There are no risks, because either you are able to consume the product or not. I’m not sure about where to put it and I’m not that familiar with it”.

(Card 5, DE)

“Lactose is only an issue if you really have to deal with that”.

(Card 5, NL)

#### 3.4.3. Placement of Nutrition Claims

In terms of the nutrition claims (Cards 19–24), these cards were placed in the appropriate expert typology group (Group 6) by a relatively low number of participants (average 42%) when compared to the frequency of the more detailed Article 14a disease risk reduction claims or Article 14b children’s claims (Figure 7). Nutrition claims were also generally less frequently placed in their appropriate expert typology groups when compared to some of the Article 13 general function claims, particularly those relating to psychological/behavioural functions (Article 13b) and those relating to weight loss/satiety (Article 13c). If the majority of participants could relatively easily place the Article 13 and Article 14 claims in their appropriate groups it raises the question of why they were less able to do this for the nutrition claims?

By exploring in more detail which groups participants placed these nutrition claims in by country, combined with the qualitative data they provided, it is possible to develop a deeper understanding of how this particular group of claims were perceived.

**Figure A1:**
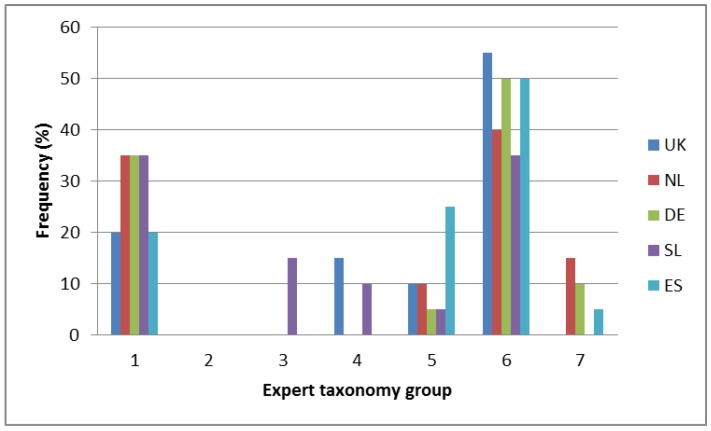
Frequency of structured sort placement of nutrition claims per country—‘Rich in vitamin C’ (Card 19).

**‘Rich in vitamin C’ (Card 19):** When not assigned to the appropriate expert typology Group 6, Card 19 ‘Rich in vitamin C’ was assigned by just over a third of participants in The Netherlands, Germany and Slovenia, and a fifth of participants in the UK and Spain to expert typology Group 1 (Appendix A, Figure A1), indicating that across all countries some participants perceived this claim to be a general function claim. However, a small number of participants from each country assigned this claim to expert typology Group 5 (Children’s development and health), although this was more pronounced in Spain. This reflected the salience of the construct ‘Relevance’ seen in the previous free sorting task whereby these participants perceived the importance of vitamin C for children as the main driver for categorising the claim and placed it in the Article 14b typology group. A number of participants from the UK and Slovenia also placed this claim in Group 4, indicating that they perceived this claim to be more appropriately categorised as a disease risk reduction claim. A small number of Slovenian participants placed this card in Group 3 perceiving it to be most relevant for weight control in some way, possibly due to vitamin C’s connection with fresh fruits and vegetables in the context of a healthy diet.

**Figure A2:**
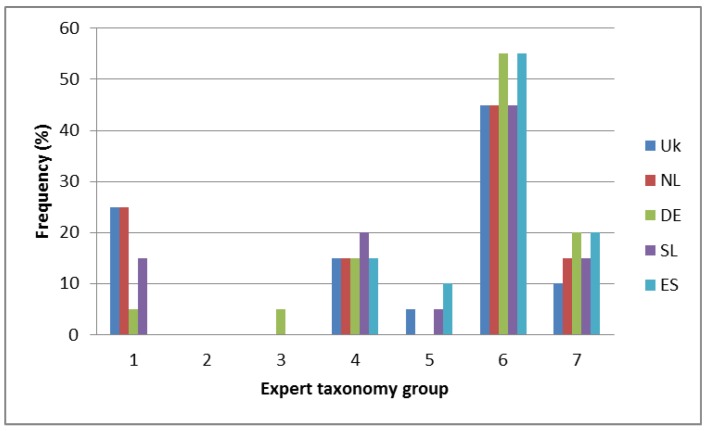
Frequency of structured sort placement of nutrition claims per country—‘Naturally low in sodium’ (Card 20).

**‘Naturally low in sodium’ (Card 20):** Card 20, ‘Naturally low in sodium’, was, on average, only placed in the appropriate expert typology Group 6 by just under 50% of the total sample; a quarter of participants in the UK and The Netherlands perceived this claim to be either a general function claim and placed it in Group 1 (Appendix A, Figure A2). Across all the countries a number of participants perceived this claim to be a disease risk reduction claim by placing it in Group 4. With this claim there was increased use of Group 7 ‘Don’t know’ (Appendix A, Table A3). Throughout the study, a number of participants expressed a certain amount of confusion and lack of familiarity with ‘sodium’ as a nutrient and this may have impacted on their ability to place this card in the structured sorting task. Reflecting on the free sorting task, this reinforces the importance of familiarity with the nutrient in a claim if consumers are going to be able to make sense of it. Whilst many people are familiar with the term ‘salt’ referring to ‘sodium’ appears to be confusing for some.

“Sodium is good for the heart”.

(DE)

“Sodium? What does it mean?”.

(SL)

“Naturally low in sodium. Means nothing to me, again it’s just a chemical I know nothing about”.

(UK)

**Figure A3:**
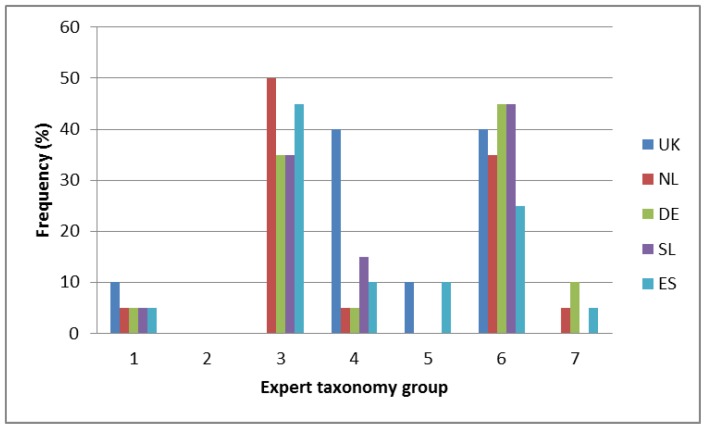
Frequency of structured sort placement of nutrition claims per country—‘Fat-free’ (Card 21).

**‘Fat-free’ (Card 21):** This card was assigned on average by 38% of participants in the Netherlands, Germany, Slovenia and Spain to Group 3, i.e., general function claims referring to slimming or weight control (Appendix A, Figure A3). 

**Figure A4:**
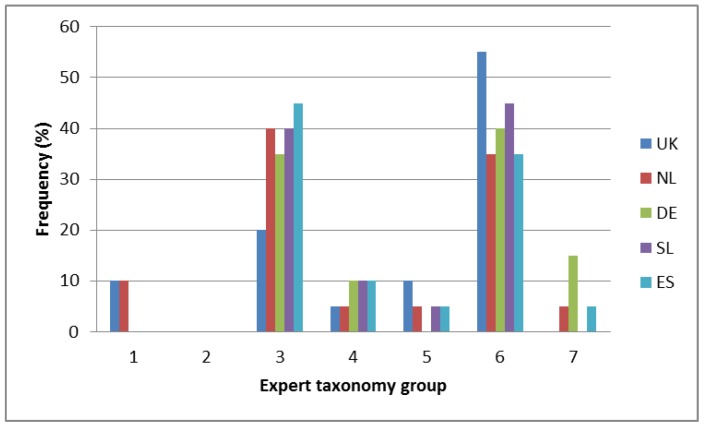
Frequency of structured sort placement of nutrition claims per country—‘No added sugar’ (Card 22).

**‘No added sugar’ (Card 22):** This card was most frequently placed in Group 3 when not assigned to Group 6, again indicating that a number of participants across all the countries perceived this claim to be a general function claim associated with slimming or weight control (Appendix A, Figure A4). A small number of participants perceived this claim to be a disease risk reduction claim or a claim of specific relevance to children.

**Figure A5:**
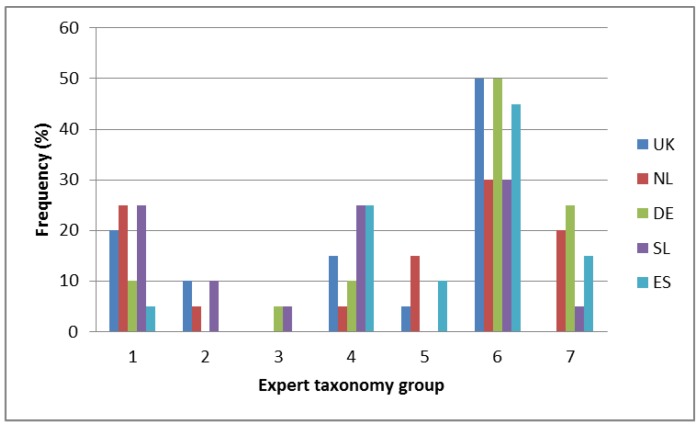
Frequency of structured sort placement of nutrition claims per country—‘Source of omega-3’ (Card 24).

**‘Source of omega-3’ (Card 24):** The structured sorting of this card demonstrated an even wider variety of opinions regarding the appropriate group heading for this claim (Appendix A, Figure A5). Whilst the majority categorised this claim as being a nutrition claim assigning it to Group 6, a number of participants assigned this claim to Group 1. This was more pronounced in the Netherlands, Slovenia and the UK than in Germany or Spain. Some participants in the UK, Slovenia and one in the Netherlands categorised this claim as Group 2 using knowledge from previous experience of the link between Omega-3 and brain function to elaborate on the information given. A small number of participants assigned this card to Group 5 based on their perception of its importance for children’s development demonstrating again the impact of ‘Relevance’ as a construct for consumers when making sense of these types of claims.

**Figure A6:**
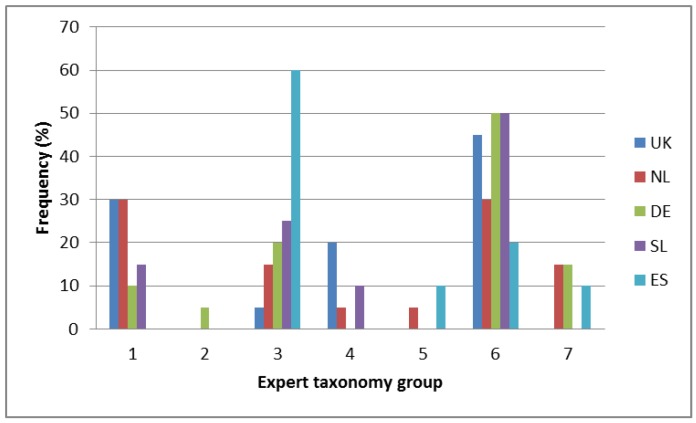
Frequency of structured sort placement of nutrition claims per country—‘Contains wholegrain’ (Card 23).

**Figure A7:**
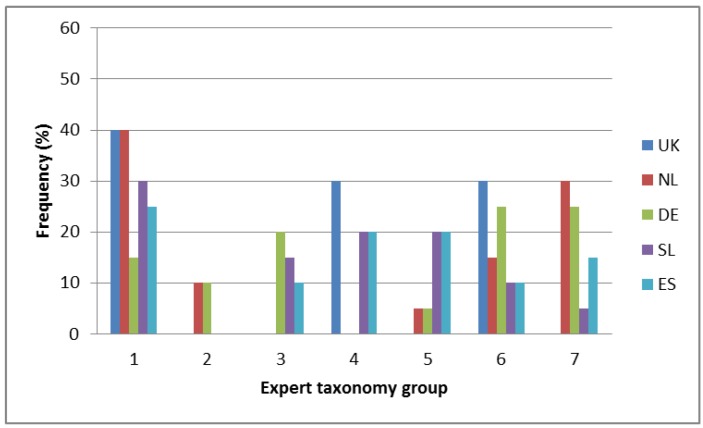
Frequency of structured sort placement of nutrition claims per country—‘One of your 5 a day’ (Card 25).

**‘Contains wholegrain’ and ‘One of your 5 a day’ (Cards 23 and 25):** Participants appeared to be more likely to attribute the ‘One of your 5 a day’ claim as a general function claim (Group 1) than they were for the ‘Contains wholegrain’ claim (Appendix A Figure A6 and Figure A7). In particular, in Spain, ‘Contains wholegrain’ was more likely to be placed in Group 3 (General function claims; Slimming, weight control and satiety) indicating that they perceived this claim to relate in some way to dieting. In contrast, ‘One of your 5 a day’ was more likely to be placed in group 5 (Children’s health and Development claims) than the ‘Contains wholegrain’ claim in Spain and Slovenia suggesting a perceived importance for children’s health and development with this particular claim. 

#### 3.4.4. Reflecting on the Expert Typology Group Headings and Structured Sorting Task

Overall, participants indicated that they found the structured sort task easier than the free sorting task. However, a small number of participants indicated that they found it more difficult than free sorting due to the constraints of the predefined categories or the complexity of many of the structured sorting group heading descriptions. On the whole, it was the definition for Group 6, the nutrition claims group, that posed the greatest difficulty for participants to interpret. In addition, participants found it difficult to distinguish between the general function claims referring to the role of a food or food constituent in the growth, development and functions of the body (Group 1) and the nutrition claims group (Group 6). This was clearly reflected in their lack of ability to place the nutrition claims appropriately in Group 6 and the tendency for some participants to place the nutrition claims in Group 1.

“Group 6 is difficult to understand; sorting was ok, but the expert terms were too difficult”.

(DE)

*“The description of group 6 was very complex/difficult to understand. This task was quite easy because it was the same* [stimuli] *cards as before so I already knew them”.*

(NL)

“Placing into group 6 was hard since if you take a little different look it would be possible to place almost all cards from group 6 into group 1. With group 4 I had problem because I had to decide whether it suggests an illness or not”.

(SL)

“The task is very difficult because it takes time to understand the differences between the groups. Group 1 and group 6 are very similar. I could not understand some words. It was very difficult to classify”.

(ES)

Participants typically found it easier to understand the structured sort heading groups relating to disease risk reduction claims (Group 4), children’s development and health (Group 5) and general function claims related to slimming and weight control (Group 3); again, this was reflected in the participant’s ability to place the appropriate claims in these groups (Figure 7).

“Groups 6 and 1 are confusing; groups 2, 3, 4 and 5 are easier”.

(UK)

“The categories to do with children and losing weight were the easiest, category 6 was difficult to understand and vague enough that you could almost put anything in there you couldn’t think where else to put”.

(UK)

“This task seems to me as the logical continuation of what we have been doing before. For me the most difficult was group 6; I had to read it a couple of times to grasp the meaning. The easiest was the group with children. Some groups are similar, so a single card could be placed in one or another group”.

(SL)

Some participants expressed difficulty with Group 2, claims describing or referring to psychological and/or behavioural functions. In addition, participants indicated that many of the claim stimuli could in their opinion be placed in more than one group.

“Some cards could have been classified in more than one group”.

(ES)

“Some cards could be placed in 2 groups, which made it difficult. For example the card about walnuts could belong to more than one group. The group with children was easy to me”.

(NL)

## 4. Discussion

### 4.1. Factors Impacting Consumer Acceptance of Health and Nutrition Claims

The results from this study suggest that consumers’ ability to process health and nutrition claims is impacted by a range of factors, but primarily whether the nutrient or substance is recognisable to them as being relevant or important to food or health in some way. When a claim refers to an unfamiliar nutrient, they appear to find the claim less understandable or credible.

“Doco-something, I’ve never heard of it” “I don’t understand this so if they would advertise with it I wouldn’t be convinced”.

(NL)

“They’re statements that are true but I worry about what they mean by barley beta glucans and plant steroids and plant sterols. I have no idea what they are, they could be plant fibres, plant sterols, plant steroids, I’m struggling to think what they might be”.

(UK)

“There are health claims which I cannot understand. I am not a biologist, who would know all these nutrients/substances”.

(SL)

“Plant sterols and plant stanol esters also belong to the second category. You may have noticed that I am not a chemist and do not know all these terms”.

(DE)

Also of importance in terms of consumer understanding and acceptance of health and nutrition claims, is whether the claim is recognisable as being relevant for them as an individual or for other specific population groups, and this construct was clearly reflected in the free sort strategies utilised by many of the participants across all the countries.

“Cholesterol level is for older people”.

(DE)

“Card 1, I do not know where to classify but it is also important for kids”.

(ES)

“I know that, because I also suffer from a high blood pressure”.

(DE)

“Decrease of tiredness, that is appealing because I’m tired”.

(NL)

“I’m not interested in cholesterol because I feel there is no danger for me yet”.

(SL)

It was also recognised by many participants in both their free sorting strategies and the qualitative data they provided that some of the claims presented to them lacked a stated benefit, function or effect whilst other claims did include this information and in some cases linked the nutrient with a disease.

“The ingredient is mentioned here but also there is an effect of each ingredient mentioned”.

(various cards, DE)

“No added sugar or fat-free, or rich in vitamin C, or source of Omega-3, contains wholegrain or naturally low in sodium, one of your 5 a day…. It is assumed, that the consumer knows their effects”.

(DE)

“This information does not say the benefits it provides.”

(Card 25, ES)

“The card about walnuts refers to how the product improves something”

(Card 6, NL)

“Of course, you could say something like: little sugar is good for diabetics. But that fact has not been mentioned in this claim”.

(Card 22, DE)

“One can recognize diseases here”.

(Card 12, DE)

However, it would appear to be less important for consumers if the stated function or benefit is omitted from a claim when the nutrient or substance in the claim is familiar to them, as they demonstrated that they are able to activate knowledge from previous experience to elaborate on the information given and decide based on this whether they perceive the claim to be beneficial to health, relevant for them or even credible. This process of ‘spreading activation’ suggests that claim statements have the ability to promote inferences that go beyond what is actually stated [31,32], although these inferences are not necessarily always correct.

“I don’t really understand these but can relate them to existing knowledge enough to take seriously, though I don’t think they’re relevant to me personally”.

(SL)

“I recently started to use vitamin B12 because someone pointed out to me that it works really well against Parkinson disease”.

(NL)

“The salt one, reducing salt, I know that’s supposed to be really good for you because it helps reduce your blood pressure”.

(UK)

“Contains wholegrain – if you eat that regularly, the risk of getting diseases is decreased.”

(DE)

“Walnuts are good for the nerves”.

(NL)

“Wholegrain, it is good for weight loss”.

(ES)

“Omega 3 is for brain, I mean not really for a brain, it is to some extent connected with problems in the stomach and problems with thought. I don’t know how to say... also fatigue, it is all connected”.

(SL)

“Sodium is good for the heart”.

(DE)

“I’ve heard this somewhere that too much calcium in the body may not affect your bone structure, but it might affect your stomach and that, you know, having too much calcium”.

(UK)

One might suggest, therefore, that the inclusion of a stated function or benefit in the claim when an unfamiliar nutrient is present may help consumers to process claims, or perhaps even minimise any potential incorrect inferences being made by consumers when a nutrient is familiar. However, our results demonstrate that by increasing the perceived level of complexity of the claim, lengthening the text or including more scientific language there is the potential to make the claim less appealing overall for many of the participants.

“The short and clear claims I find most appealing. I have to think really hard about the other claims”.

(NL)

“I believe that on these cards (cards 4 & 14) they could reduce the amount of information written”.

(SL)

In addition, a number of participants across all the countries indicated that they would be unlikely to engage with the more detailed, complex claims when shopping.

“Such long texts are obstructive. After all I want to go shopping and not reading novels”.

(DE)

“If there is a lot of writing someone who is the customer in the shop will not read it, because he does not have patience to read”.

(SL)

Conversely, some participants expressed the desire for more information, or perhaps better clarity, with respect to the nutrition claims, particularly those related to fat and sugar.

“If you find on card sentence “without fat” it does not tell you a lot; it could be good or bad for your health”.

(SL)

“We know that vitamin C is healthy, but the claim does not say it is healthy. But vitamin C is healthy. This claim does not say that it is good or bad”.

(NL)

“It’s more complicated, just “fat-free” is a bland statement”.

(UK)

Despite the shorter, less complex nutrient claims being generally described more favourably in terms of complexity, due to the lack of a stated function or benefit these claims were described by some as more likely to be promotional recommendations which invoked acceptance and credibility issues, particularly in the UK and this was also echoed in some of the other countries.

“This is just a promotion to make us buy [the product]”.

(SL)

*“It is interesting to me that when I eat an orange it is rich in vitamin C. But if that is stated on a package I’m not sure if that’s really true. These things have to be stated on products in order to make them sell, it seems*”.

(NL)

### 4.2. Consumer-Derived Typology for Nutrition and Health Claims

Our results suggest that depending on the associative networks consumers have previously formed between familiar nutrients and health benefits, they may not consciously differentiate between a nutrition claim and a health claim in the way that regulatory experts do. While this is in line with previous research [8,11,12,13,14] the value of our research is that the MSP methodology provides rich qualitative data across a wide range of claims, providing an explanation as to why this might occur and revealing where there is greatest potential for consumer misunderstanding. 

The free sorting results suggest that when categorising claims, consumers do not appear to differentiate between ‘Article 13a General function’ claims relating to growth development and functions of the body and ‘Article 14 Disease risk reduction’ claims in the way that regulatory experts do; although, in the structured sorting they were more likely to place the disease risk reduction claims under the appropriate expert typology group than they were for the Article 13a General Function claims. 

Driven by how participants across the five countries categorised and made sense of the various nutrition and health claims presented in this study, we propose a typology based on three key dimensions:

1. Familiarity with the nutrient, substance or food stated in the claim.
2. Statement type in terms of its simplicity/complexity.
3. Relevance of the claim, either personally or for a stated population group.

**Familiarity with the nutrient, substance or food stated in the claim:** It has been suggested that consumer perceptions of health claims are often driven by prior beliefs about a food product or nutrient rather than by the information provided within a claim [31]. Whether a claim contains a stated benefit or function appears to be of less importance to the consumer if they are familiar with the nutrient or functional ingredient since they appear to be able to draw on an associative network of stored knowledge and associations [32], which they then use to make sense of the claim. It would appear from the way in which some of the claims were assigned by participants in the structured sorting task this spreading activation [33,34] can also lead to associations being made between a claim and a general function or the reduction of disease risk even when these were not stated in the claim. Therefore, consumer understanding or misunderstanding of nutrition and health claims, whilst affected by a number of factors, appears to be impacted primarily by their familiarity with the nutrient or substance within the claim.

“This additional information is nonsense … because everybody knows that calcium is good for bones”.

(Card 1)

**Statement type in terms of its simplicity/complexity:** In-line with previous research [13,35], our results also demonstrate that consumers perceive short and simple claims more favourably, are unlikely to engage with detailed information on the product packaging whilst shopping and are unlikely to perceive information associated with an unfamiliar nutrient positively, regardless of how detailed it is. In addition, expression of the more detailed general function claims or disease risk reduction claims utilising ‘scientific’ or ‘regulatory’ language is a problem for many consumers. Therefore, their preformed associative networks are unlikely to be formed or corrected by increasing the level or scientific basis of the information placed on the food packaging in the form of a complex claim statement. Moreover, recent research has shown that adding information to the claim does not necessarily lead to improvements in adequate understanding [36].

**Relevance of the claim, either personally or for a stated population group:** In terms of consumers’ ability to assign claims to the expert typology from the NHCR within our study, it would appear that this is facilitated when the claim is deemed to be personally relevant by the consumer. Previous research has suggested that motivation to process a claim into meaningful understanding is an important factor [8] as is how easily consumers can link the information in the claim to that which they have previously stored in their memory [9]. In addition, Dean et al. (2012) demonstrated that relevance has a strong influence on perceptions of personal benefit and willingness to buy products with health claims [37]. Therefore, relevance would appear to be a key factor in influencing consumer understanding, but also whether a claim is perceived favourably or not.

### 4.3. Policy Implications

By considering the various nutrition and health claims according to the proposed three key consumer-derived dimensions, regulatory bodies concerned with appropriate consumer understanding of health claims and stakeholders concerned with promoting consumer acceptance of health claims, can perhaps gain a deeper insight into this domain from a consumer perspective.

In terms of promoting consumer acceptance of health and nutrition claims, any claim classified as 1a/2a/3a by the proposed typology (Table 5) is likely to be the most favourably received by consumers in that it refers to a familiar nutrient, substance or food for which the consumer has previous knowledge to draw upon. That is to say, it states the claim in a nutrient content format only, and resonates with the consumer because they perceive it to be personally relevant. In contrast, claims classified as 1b/2b/3c by this typology are likely to be the least favourably received by consumers in that they contain an unfamiliar nutrient, are complex and not easily attributable in terms of relevance to oneself or a specific population group. This perhaps explains why the claim on Card 8 ‘DHA contributes to normal brain function’ was so poorly perceived by our study participants. 

Whilst claims classified by this typology as 1a/2a/3a are the most likely to be positively perceived by consumers it should be recognised that from a regulatory perspective, they also have the greatest potential to promote the process of spreading activation and possibly even the generation of incorrect inferences in consumers. The degree to which this may occur is obviously dependent on the associative networks that a consumer has previously established in relation to a particular nutrient. 

In line with Ausubel’s theory of Meaningful Learning [38], the results from our study suggest that both the associative networks and beliefs that consumers have previously developed in relation to nutrients/substances and their relationship with health outcomes, are key drivers to the way in which health claims are interpreted and understood. They also provide further evidence that consumers do not consciously differentiate between a nutrition claim and a health claim in the way that regulatory experts envision they should do. Particularly, when nutrients/substances in the claim are familiar and personally relevant there is the potential for consumers to ‘upgrade’ the former for the latter simply based on their network and prior beliefs, as opposed to what is actually stated in the claim. From a regulatory point of view, if the actual format of the claim, that is, whether it is a detailed risk reduction claim or a simple nutrition claim, is of less importance to the consumer when they have a preformed associative network for the nutrient or substance in the claim, it is then imperative that the associative networks consumers draw upon are correct and well-informed. 

Our results support previous research findings that consumers perceive short and simple claims more favourably [13,14,35], are unlikely to engage with detailed information on the product packaging whilst shopping and, are unlikely to perceive information associated with an unfamiliar nutrient positively, regardless of how detailed it is. In addition, they suggest that expression of the more detailed general function claims or disease risk reduction claims utilising ‘scientific’ or ‘regulatory’ language is a problem for many consumers. Therefore, these associative networks are unlikely to be formed or corrected by increasing the level or scientific basis of the information placed on the food packaging in the form of a complex claim statement. It is also important to recognise, at this point, that the removal of a claim from the packaging of a product or product category is unlikely itself, to result in consumers spontaneously readdressing their preformed associative networks regarding the benefits of that product or product category. For example, in the UK, it has been suggested that yoghurt sales have not been significantly impacted as a result of the removal of digestive health claims due to successful repositioning in terms of positive lifestyle and general wellbeing. However, it has also been suggested that this could be due, in some part, to an ‘echo chamber’ of embedded digestive health benefits within consumers previously formed associative networks [39].

Furthermore, how aware are consumers of the relatively recent changes to the health claims legislation? It is fair to suggest that there has not been any comprehensive or structured communication to consumers on the differences between the various levels of health claims that are now permitted/not permitted and what they really mean. Regulatory bodies and those stakeholders concerned with fostering appropriate understanding of health claims in consumers should, therefore consider employing strategies to impact on this awareness in consumers, and also to impact on the associative networks consumers have previously made around particular nutrients in the nutrition and health claim domain. 

For more familiar nutrients or functional ingredients where strong associative networks have been previously formed, but the claims are no longer legally allowed by the regulations, there is a need to re-educate the consumer appropriately. Similarly, for new functional ingredients, or less familiar nutrients, where associative networks have not been previously formed, there is an opportunity to educate the consumer appropriately, thus making claims for these functional ingredients potentially more appealing to consumers. 

When products containing relatively unfamiliar nutrients or new functional ingredients are developed, it has been previously recognised that these need to be supported by an effective communication strategy to inform consumers of their function and benefits [35]. In the commercial world, establishing associative networks in relevant consumer groups is a fundamental part of an effective product marketing strategy. These strategies are usually delivered in the form of magazine editorials, television advertising and more recently social media and other consumer resources accessed via the internet. Once established, the associative networks formed in consumers’ minds by these mass marketing strategies are then activated via short and consumer-tailored statements on the product packaging at point of purchase. 

It is interesting to note at this point that to parallel the above commercial efforts to promote new products displaying health claims, there appears to be no authoritative information or educational resource that is independent from industry for consumers to draw upon easily. The existing EU legislation and/or the scientific literature is of little use to the lay consumer in helping them to form new, or even correct, their existing associative networks for the nutrients, functions and benefits within the health claims arena both past and present. Since two of the three main constructs identified in the proposed typology—‘familiarity’ and ‘relevance’—are constructs with individual differences, the situation is further complicated by the fact that different consumers are likely to receive the same claims differently based on their established network and beliefs.

## 5. Conclusions

The outcomes of this research suggest important recommendations for improving the communication of healthier food choice. Our results demonstrate that consumers may not consciously differentiate between a nutrition claim and a health claim in the way that is expected by the NHCR regulation. The richness of the qualitative data provided by the MSP methodology across a wide range of claims provides an explanation as to why this might occur and reveals where there is the greatest potential for consumer misunderstanding. 

Our results confirm that consumers are likely to receive the same claims differently based on their pre-established networks and beliefs. Regulators need to consider providing resource(s) to help consumers re-establish appropriate networks and beliefs associated with the more familiar nutrients/substances thus helping them to understand and respond appropriately both to the types of health claims now appearing on packaging and to their specific wording. 

Similarly, for new functional ingredients or less familiar nutrients, where associative networks have not yet formed, there is an opportunity to educate the consumer appropriately, potentially making claims for these functional ingredients more favourably received by consumers. Indeed a small number of participants faced with an unfamiliar nutrient or benefit spontaneously expressed the desire to search for information in order to educate themselves, citing ‘Google’ as their default means of accessing this information.

“Now the Vitamin B12 topic appears again, I don’t know anything about the metabolism of homocysteine, but I will Google it at home.”

“Haven’t the faintest idea what that means, other than that it’s going to help my cholesterol, but what MUFA and PUFA is, I haven’t the foggiest idea. It seems to be a completely complicated statement, but should I go onto Google and find out for real?”

## 6. Limitations and Future Research Opportunities

Despite the small sample size, the value of the type of information gathered from this study is that it is difficult to capture in larger questionnaire based quantitative studies. However, it should be noted that this study was not concerned with testing the effectiveness of nutrition and health claims in driving product choice, its purpose was to elicit semi-structured qualitative data to gain a deeper understanding of how consumers describe and differentiate the various nutrition and health claims. Therefore, as an exploratory study based on forced exposure, participants may have been more likely to be sceptical of the claims shown to them than they would be in a real-world shopping setting, since this often does not involve careful inspection of the claims present on pack. In addition, the stimuli used in the study were stripped of any contextual factors such as brand or packaging imagery, which are known to impact on consumer perceptions and choice. For the purposes of developing a consumer-derived typology of the claims themselves it was necessary to exclude these possible confounding factors from the study designs. 

Future research could seek to build on the outcomes of this study across a more representative population sample to explore more fully these important contextual factors and their impact on communication of appropriate healthier food choices. There is also potential to explore the outcomes of this research within the framework of expert theories such as Meaningful Learning and other consumer information models to establish how well these consumer-derived constructs fit with these models. Further research is also needed to explore consumer understanding in more ecologically valid environments. 

## Figures and Tables

**Table 1:** Sociodemographic characteristics of participants by country.

	Germany	Netherlands	Slovenia	Spain	UK	Total
	*n* = 20	*n* = 20	*n* = 20	*n* = 20	*n* = 20	*N* = 100
Sex						
Male	10	10	10	10	10	50
Female	10	10	10	10	10	50
Age						
18–34 years	9	6	5	4	3	27
35–49 years	3	6	5	8	4	26
50–64 years	5	4	6	5	9	29
65+ years	3	4	4	3	4	18
Highest education						
Primary	0	0	3	3	0	6
Secondary ^1^	16	12	10	12	15	65
University	4	8	7	5	5	29

^1^ This subgroup includes participants who completed further vocational education postsecondary level.

**Table 2:** Health claim and nutrition claim stimuli by expert typology and health relationship.

Stimuli No.	NHCR Expert Typology	Claim Wording on Stimuli Card
1	13(1)a	Calcium is needed for the maintenance of normal bones.
2	13(1)a	Vitamin B12 contributes to normal homocysteine metabolism.
3	13(1)a	Reducing consumption of sodium contributes to the maintenance of normal blood pressure.
4	13(1)a	Replacing saturated fats with unsaturated fats in the diet contributes to the maintenance of normal blood cholesterol levels (MUFA and PUFA are unsaturated fats).
5	13(1)a	Live cultures in yoghurt or fermented milk improve lactose digestion of the product in individuals who have difficulty digesting lactose.
6	13(1)a	Walnuts contribute to the improvement of the elasticity of blood vessels.
7	13(1)b	Zinc contributes to normal cognitive function.
8	13(1)b	DHA contributes to maintenance of normal brain function
9	13(1)b	Pantothenic acid contributes to the reduction of tiredness and fatigue.
10	13(1)c	Substituting one daily meal of an energy restricted diet with a meal replacement contributes to the maintenance of weight after weight loss.
11	13(1)c	Glucomannan in the context of an energy restricted diet contributes to weight loss
12	14(1)a	Sugar-free chewing gum helps reduce tooth demineralization. Tooth demineralization is a risk factor in the development of dental caries.
13	14(1)a	Barley beta glucans have been shown to lower/reduce blood cholesterol. High cholesterol is a risk factor in the development of coronary heart disease.
14	14(1)a	Plant sterols and plant stanol esters have been shown to lower/reduce blood cholesterol. High cholesterol is a risk factor in the development of coronary heart disease.
15	14(1)b	Docosahexaenoic acid (DHA) intake contributes to the normal visual development of infants up to 12 months of age.
16	14(1)b	Calcium and vitamin D are needed for normal growth and development of bone in children.
17	14(1)b	Iron contributes to normal cognitive development of children.
18	14(1)b	Essential fatty acids are needed for normal growth and development of children.
19	Nutrition claim	Rich in vitamin C.
20	Nutrition claim	Naturally low in sodium.
21	Nutrition claim	Fat-free.
22	Nutrition claim	No added sugar.
23	-	Contains wholegrain.
24	Nutrition claim	Source of Omega-3.
25	-	One of your 5 a day. (Experts recommend you eat 5 portions of fruit and vegetables every day. That is 5 portions in total, not 5 portions of each.)

**Table 3:** Structured sort headings and associated stimuli cards.

Structured Sort Heading No.	NHCR Expert Typology	Heading Wording on Card	Associated Stimuli Cards
1	13(1)a	Claims describing or referring to the role of a food or food constituent in the growth, development and functions of the body.	1–6
2	13(1)b	Claims describing or referring to psychological and/or behavioural functions.	7–9
3	13(1)c	Claims describing or referring to slimming or weight control or; a reduction in the sense of hunger, an increase in the sense of satiety, the reduction of the available energy from the diet.	10–11
4	14(1)a	Claims stating, suggesting or implying that the consumption of a food or food constituent significantly reduces a risk factor in the development of a human disease.	12–14
5	14(1)b	Claims relating to children’s development and health.	15–18
6	Nutrition claim	Claims stating, suggesting or implying that a food has particular beneficial nutritional properties due to; the energy (calorific value) it provides, at a reduced or increased rate, or does not provide and/or the nutrients or other substances it contains, contains in reduced or increased proportions or does not contain.	19–22, 24
7	-	Don’t know.	

**Table 4:** Mean scores for background variables.

Variable	Germany (DE)	Netherlands (NL)	Slovenia (SL)	Spain (ES)	United Kingdom (UK)
General Health Interest	3.19	3.44	3.59	3.46	3.31
Need for Cognition	3.32	4.30	3.68	3.24	3.73
Faith in Intuition	3.78	3.64	3.71	3.49	3.56
Subjective knowledge of health claims	3.17	3.23	3.67	3.52	3.52
Motivation to process health claims	3.73	3.48 ^1^	3.78	4.25 ^1^	3.55

^1^ One-way ANOVA, *F*(4, 95) = 2.957; *p* = 0.024, all other comparisons nonsignificant.

**Table 5:** Proposed typology dimensions for nutrition and health claims.

Dimension		a	b	c
1	Familiarity with nutrient, substance or food	Familiar	Unfamiliar	-
2	Statement type	Simple—refers to nutrient, substance or food only (i.e., nutrient content)	Complex—refers to both nutrient, substance or food and benefit	-
3	Relevance	Personally relevant	Population group relevance stated	No relevance

## Appendix A

**Table A1:** Categories of constructs used in free sorting for all countries combined.

Sort strategy Category/Constructs	Free Sort Number					Total Frequency
	1	2	3	4	5	
Information contained in claim: Nutrient/health condition or outcome/function/purpose/benefits (includes reference to consequences/risk communication)	24	24	9	4	0	61
Types of statements: Complexity/length/information levels/specific vs. general information/expertise required vs. user friendliness	22	14	6	3	3	48
Relevance: Personal/target groups/appeal	14	15	5	3	1	38
Mixed sort—no dominant construct	10	6	2	1	1	20
Understanding/confusion	7	4	5	0	0	16
Natural/artificial: Scientific vs. naturally occurring/healthful vs. not healthful/processed vs. not processed	5	3	3	0	0	11
Importance	5	3	0	0	1	9
Credibility: Believability/measurability/substantiation level/trust/agreement	3	3	1	1	1	9
Food: Food group, food supplement	4	1	1	3	0	9
Familiarity: Popularity/known or not known	3	0	1	1	0	5
Position on pack: Front vs. back	2	1	2	0	0	5
Effect: Duration/direction	1	1	2	0	0	4
Positive vs. negative message: Warning/Inclusion vs. absence	0	1	1	1	1	4
Plant- vs. animal-based	0	1	0	0	1	2
Meal relevance	0	0	2	0	0	2
Clarity	0	1	0	0	0	1
Need	0	1	0	0	0	1
Total Free Sorts	100	79	40	17	9	245

**Table A2:** Categories of constructs used in free sorting per country.

Sort strategy Category/Constructs	Country Frequencies					Total Frequency
	DE	NL	SL	ES	UK	
Information contained in claim: Nutrient/health condition or outcome/function/purpose/benefits (Includes reference to consequences/risk communication)	2	8	4	5	5	24
Types of statements: Complexity/length/information levels/specific vs. general information/expertise required vs. user friendliness	8	3	4	3	4	22
Relevance: Personal/target groups/appeal	3	2	2	2	5	14
Mixed sort—no dominant construct	0	2	1	7	0	10
Understanding/confusion	1	2	1	0	3	7
Natural/artificial: Scientific vs. naturally occurring/healthful vs. not healthful/processed vs. not processed	1	2	1	1	0	5
Importance	3	0	1	1	0	5
Credibility: Believability/measurability/substantiation level/trust/agreement	0	0	1	0	2	3
Familiarity: Popularity/known or not known	2	0	1	0	0	3
Food: Food group, food supplement	0	0	4	0	0	4
Position on pack: Front vs. back	0	0	0	1	1	2
Effect: Duration/direction	0	1	0	0	0	1

**Table A3:** Frequency of stimuli cards placed in Structured Sort Group 7 “Don’t know” by country.

Card ^1^	Claim Wording	Percentage (%)					
		DE	NL	SL	ES	UK	Total
20	Naturally low in sodium.	20	15	15	20	10	80
25	One of your 5 a day.	25	30	5	15	0	75
2	Vitamin B12 contributes to normal homocysteine metabolism.	25	15	5	20	5	70
24	Source of Omega-3	25	20	5	15	0	65
7	Zinc contributes to normal cognitive function.	10	10	10	15	0	45
9	Pantothenic acid contributes to the reduction of tiredness and fatigue.	30	5	5	5	0	45
23	Contains wholegrain.	15	15	0	10	0	40
19	Rich in vitamin C.	10	15	0	5	0	30
8	DHA contributes to maintenance of normal brain function.	20	0	5	0	0	25
12	Sugar-free chewing gum helps reduce tooth demineralisation. Tooth demineralisation is a risk factor in the development of dental caries.	10	0	5	0	10	25
22	No added sugar.	15	5	0	5	0	25
3	Reducing consumption of sodium contributes to the maintenance of normal blood pressure.	5	0	5	10	0	20
5	Live cultures in yoghurt or fermented milk improve lactose digestion of the product in individuals who have difficulty digesting lactose.	10	5	0	0	5	20
21	Fat-free.	10	5	0	5	0	20
4	Replacing saturated fats with unsaturated fats in the diet contributes to the maintenance of normal blood cholesterol levels (MUFA and PUFA are unsaturated fats).	0	5	0	10	0	15
11	Glucomannan in the context of an energy restricted diet contributes to weight loss.	5	0	0	5	0	10
15	Docosahexaenoic acid (DHA) intake contributes to the normal visual development of infants up to 12 months of age.	5	0	5	0	0	10
13	Barley beta glucans has been shown to lower/reduce blood cholesterol. High cholesterol is a risk factor in the development of coronary heart disease.	5	0	0	0	0	5
14	Plant sterols and plant stanol esters have been shown to lower/reduce blood cholesterol. High cholesterol is a risk factor in the development of coronary heart disease.	5	0	0	0	0	5

^1^ Stimuli cards 1, 6, 10, 16, 17 & 18 were not placed in the “Don’t know” structured sort category by any participant.

## References

[1] Habib S.H., Saha S. (2010). Burden of non-communicable disease: Global overview. Diabetes Metab. Syndr..

[2] Cowburn G., Stockley L. (2005). Consumer understanding and use of nutrition labelling: A systematic review. Public Health Nutr..

[3] Van Kleef E., Dagevos H. (2015). The growing role of front-of-pack nutrition profile labeling: A consumer perspective on key issues and controversies. Crit. Rev. Food Sci..

[4] (2011). Commission Regulation (EC) 1169/2011 on the Provision of Food Information to Consumers. Off. J. Eur. Union.

[5] Nutrition and Health Claims (2015). Evaluation of A) Regulation (EC) no 1924/2006 on Nutrition and Health Claims Made on Food with Regard to Nutrient Profiles and Health Claims Made on Plants and Their Preparations and of B) the General Regulatory Framework for Their Use in Foods. https://ec.europa.eu/food/safety/labelling_nutrition/claims/refit_en.

[6] (2006). Commission Regulation (EC) 1924/2006 of 20 December 2006 on Nutrition and Health Claims Made on Foods. Off. J. European Union.

[7] (2006). Eu Register on Nutrition and Health Claims.

[8] Grunert K.G., Scholderer J., Rogeaux M. (2011). Determinants of consumer understanding of health claims. Appetite.

[9] Nocella G., Kennedy O. (2012). Food health claims—What consumers understand. Food Policy.

[10] Roe B., Levy A.S., Derby B.M. (1999). The impact of health claims on consumer search and product evaluation outcomes: Results from fda experimental data. J. Public Policy Mark..

[11] Leathwood P.D., Richardson D.P., Strater P., Todd P.M., van Trijp H.C.M. (2007). Consumer understanding of nutrition and health claims: Sources of evidence. Br. J. Nutr..

[12] Verbeke W., Scholderer J., Lahteenmaki L. (2009). Consumer appeal of nutrition and health claims in three existing product concepts. Appetite.

[13] Williams P. (2005). Consumer understanding and use of health claims for foods. Nutr. Rev..

[14] Verhagen H., Vos E., Francl S., Heinonen M., van Loveren H. (2010). Status of nutrition and health claims in europe. Arch. Biochem. Biophys..

[15] Smith E., Medin D. (1981). Categories and Concepts.

[16] Rugg G., McGeorge P. (1997). The sorting techniques: A tutorial paper on card sorts, picture sorts and item sorts. Expert Syst..

[17] Barnett J., Breakwell G.M. (2004). The multiple sorting procedure. Doing Social Psychology Research.

[18] Kelly G. (1955). The Psychology of Personal Constructs.

[19] Adams-Webber J.R. (1970). Elicited versus provided constructs in repertory grid technique—A review. Br. J. Med. Psychol..

[20] Hodgkins C., Barnett J., Wasowicz-Kirylo G., Stysko-Kunkowska M., Gulcan Y., Kustepeli Y., Akgungor S., Chryssochoidis G., Fernandez-Celemin L., Bonsmann S.S.G. (2012). Understanding how consumers categorise nutritional labels: A consumer derived typology for front-of-pack nutrition labelling. Appetite.

[21] Wilson M.A., Breakwell G.M., Hammond S., Fife-Schaw C. (2000). Structuring qualitative data: Multidimensional scalogram analysis. Research Methods in Psychology.

[22] Lingoes J., Lingoes J., Roskam E., Borg I. (1979). The multivariate analysis of qualitative data. Geometric Representations of Relational Data.

[23] Zvulun E., Shye S. (1978). Multidimensional scalogram analysis: The method and its application. Theory Construction and Data Analysis in the Behavioural Sciences.

[24] Canter D.V., Brown J., Groat L., Brenner M., Brown J., Canter D.V. (1985). A multiple sorting procedure for studying conceptual systems. The Research Interview: Uses and Approaches.

[25] Roininen K., Lahteenmaki L., Tuorila H. (1999). Quantification of consumer attitudes to health and hedonic characteristics of foods. Appetite.

[26] Roininen K., Tuorila H., Zandstra E.H., de Graaf C., Vehkalahti K., Stubenitsky K., Mela D.J. (2001). Differences in health and taste attitudes and reported behaviour among finnish, dutch and british consumers: A cross-national validation of the health and taste attitude scales (htas). Appetite.

[27] Moorman C., Diehl K., Brinberg D., Kidwell B. (2004). Subjective knowledge, search locations, and consumer choice. J. Consum. Res..

[28] Moorman C. (1990). The effects of stimulus and consumer characteristics on the utilization of nutrition information. J. Consum. Res..

[29] Cacioppo J.T., Kao C.F., Petty R.E., Rodriguez R. (1986). Central and peripheral routes to persuasion—An individual difference perspective. J. Personal. Soc. Psychol..

[30] Epstein S., Pacini R., DenesRaj V., Heier H. (1996). Individual differences in intuitive-experiential and analytical-rational thinking styles. J. Personal. Soc. Psychol..

[31] Sims L.S. (1999). Federal trade commission study on food health claims in advertising: Implications for nutrition education and policy. J. Nutr. Educ..

[32] Solomon M.R. (1996). Consumer Behaviour: Buying, Having and Being.

[33] Collins A.M., Loftus E.F. (1975). Spreading activation theory of semantic processing. Psychol. Rev..

[34] Anderson J.R. (1983). A spreading activation theory of memory. J. Verb. Learn. Verb. Behav..

[35] Siro I., Kapolna E., Kapolna B., Lugasi A. (2008). Functional food. Product development, marketing and consumer acceptance-a review. Appetite.

[36] Stancu V., Grunert K.G., Lahteenmaki L. (2017). Consumer inferences from different versions of a beta-glucans health claim. Food Qual. Preference.

[37] Dean M., Lampila P., Shepherd R., Arvola A., Saba A., Vassallo M., Claupein E., Winkelmann M., Lahteenmaki L. (2012). Perceived relevance and foods with health-related claims. Food Qual. Preference.

[38] Ausubel D., Andersen R.C., Ausubel D.P. (1965). A cognitive structure view of word and, concept meaning. Readings in the Psychology of Cognition.

[39] Arthur R. (2014). Smooth Transition: Yogurt Is Doing Fine without Digestive Health Claims. http://www.dairyreporter.com/Markets/yogurt-Euromonitor-digestive-health-EU.

